# Treatment of Moderately Crowded Teeth Using Lingual Fixed Appliance Prepared by a Modified HIRO® Technique: A Case Report and Method Description

**DOI:** 10.7759/cureus.25077

**Published:** 2022-05-17

**Authors:** Jehad M Kara-Boulad, Ahmad S Burhan, Mohammad Y Hajeer, Tarek Z. Khattab, Fehmieh R Nawaya, Rabab Al-Sabbagh

**Affiliations:** 1 Department of Orthodontics, Damascus University, Damascus, SYR; 2 Department of Orthodontics, University of Hama, Hama, SYR; 3 Department of Pediatric Dentistry, Syrian Private University, Damascus, SYR

**Keywords:** lingual appliance, manual laboratory, virtual setup, hiro® technique, bracket transfer caps, composite pads, lingual orthodontics, 3d printing, moderate crowding of anterior teeth, fixed appliances

## Abstract

There are various manual laboratory methods available for indirect positioning and bonding of lingual brackets. The manual setup has limitations because of its complicated laboratory procedures and requires time and specialized laboratory equipment. In addition, the manual method is also prone to human errors. In this case report, a description of a new method of laboratory preparation for the indirect bonding of lingual brackets is given by merging recent advances in digital dentistry with some of the ordinary manual steps in this field. Therefore, the well-known HIRO® technique has been modified by using the three-dimensional (3D) virtual setup instead of the traditional manual setup. This method does not require the use of any specialized laboratory equipment, and it is also cost-effective for patients who cannot afford fully customized lingual appliances. In this modified technique, 3Shape Ortho Analyzer™ software (3Shape, Copenhagen, Denmark) and a 3D printer (Prusa® i3 mk3; Prusa Research, Prague, Czech Republic) were used to align the teeth three-dimensionally into their desired positions and to produce the final working printed model on which lingual brackets were placed, and transfer caps were fabricated for clinical use.

## Introduction

There has been a great interest in lingual orthodontics due to being considered the best solution for preserving esthetic appearance without minimizing the biomechanical efficiency [1]. Esthetic appearance has become influential among adult patients and is the most frequent encouraging motive for patients to request orthodontic treatment [2]. The lingual technique is relatively complicated due to the preparation of appliances, setup, indirect bonding, and rebonding of loose brackets throughout the course of treatment [3]. In addition, the lingual technique is considered the most difficult orthodontic technique requiring great manual skills and experience compared to the labial one. However, it is the only treatment that can be regarded as entirely invisible with a three-dimensional (3D) control of orthodontic tooth movement [4]. In lingual orthodontics, indirect bonding is the only recommended technique for bonding lingual brackets [5]. This is due to the variable lingual anatomy, short clinical crown height, smaller inter-bracket distance, slopped lingual surface, and tongue interference; therefore, the indirect bonding technique is pivotal for success in lingual orthodontics [6].

Several laboratory techniques have been developed for indirect positioning and bonding of lingual brackets. They can be classified into two categories; (1) the manual setup, which uses traditional dental models such as the torque angulation reference guide (TARG™), Bonding with Equal Specific Thickness (BEST), customized lingual appliance set-up system (CLASS), and the HIRO® system; and (2) the completely customized digital lingual setup which manufactured by computer-aided design/computer-aided manufacturing (CAD/CAM) technology like Incognito™ (3M-Unitek, Monrovia, California), Orapix™ system (Orapix Co. Ltd., Seoul, Korea), and WIN™ (DW Lingual Systems, Bad Essen, Germany) [7]. Manual laboratory methods are prone to errors and require manual dexterity and specialized laboratory equipment [8]. The laboratory devices for torque and angulation were fraught with inaccuracies and this may jeopardize treatment results [9].

The HIRO system is considered one of the most employed techniques as it is easy to perform, does not require the use of any specialized laboratory equipment, is less expensive, and re-bonding is very easy and accurate with the set-up and the basic archwire [10]. It uses an ideal arch form and a 0.017" × 0.025" stainless steel wire to position brackets on the final setup. The brackets are transferred directly onto the patient using rigid single-tooth transfer trays [11]. Several modifications to the HIRO system have been recommended in literature with the advent of newer materials [12]. Therefore, having an accurate setup is the most important step in the HIRO indirect bonding system to achieve a functional, esthetic, and stable occlusion [13]. These requirements can only be met if tip, torque, and in-out have been incorporated into the model setup [14].

Recently, the use of digital technologies such as intraoral digital scanners and 3D model scanners has become increasingly common, and the accuracy, validity, and reliability of using 3D digital models have been evaluated by many researchers and considered satisfactory [15]. Another important step is digital 3D printing which can be reconstructed and print the digital files by different printing technologies with acceptable accuracy [16]. The computer programs can be used with digital models to move the teeth in 3D, which helps in decreasing the number of steps in the existing manual setup and saving laboratory time [17]. However, one of the shortcomings of using digital models is that they show only the crowns without the roots, and this may affect proper root alignment with the possible adverse consequences of non-parallelism, bone fenestration, and dehiscence [18]. The widespread use of cone-beam computed tomography (CBCT) has given the ability to see the whole teeth in 3D, in addition to the wealth of knowledge in bone morphology without having the problems of distortion, magnification, and superimposition inherent with the classic 2D visualization modes [19,20]. Merging digital models and CBCT-derived information can help in the virtual treatment planning of the desired dental arches by positioning each tooth with reference to the occlusal plane and with respect to the anatomical boundaries of the face and skull [7,21].

This case report describes a treatment of a patient with lingual orthodontics using a new method of preparation for the indirect bonding of the lingual brackets by merging manual and digital techniques. Therefore, the HIRO technique has been modified in the steps related to the tooth positioning on a virtual model and in the printing of the final simulated model. In contrast, the steps related to the fabrication of transfer trays remained manual as in the original HIRO technique. So in this manuscript, an illustration of the workflow for fabricating a virtual setup is given in a way that allows the preparation of precise transfer trays (caps) for indirect bonding using dedicated software (3Shape Ortho Analyzer™; 3Shape, Copenhagen, Denmark) and a three-dimensional printer (Prusa i3 mk3; Prusa Research, Prague, Czech Republic).

## Case presentation

Diagnosis

**Figure 1:**
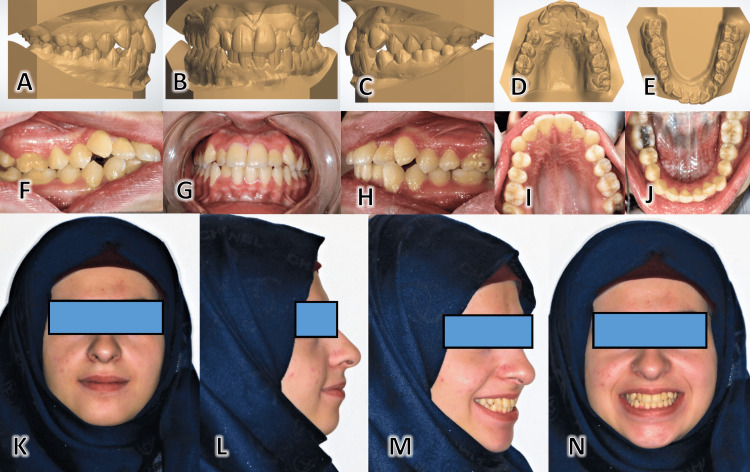
Pre-treatment patient records The plaster study models are shown from the right-hand side (A), the frontal view (B), the left-hand side (C), the occlusal view for the upper dental arch (D), and the occlusal view of the lower dental arch (E). Intra-oral photographic images of this case show the dentition from the right-hand side (F), the frontal view (G), the left-hand side (H), the occlusal view for the upper dental arch (I), and the occlusal view for the lower dental arch (J). Extra-oral photographic images included the following poses: the frontal face image at rest (K), the lateral image at rest (L), the 45-degree image with a smile (M), and the frontal face image at smile (N).

A twenty-year-old female patient presented at the Orthodontic Clinic of Damascus University in Damascus, Syria, to assess her malocclusion, requesting treatment of her crowded teeth, indicating her preference for a short and aesthetic treatment solution (Figure 1).

Extraoral examination showed a good facial proportion with a convex profile, competent lips, and smile line, which followed the curvature of the lower lips. Intraoral and dental cast examinations revealed that the patient had a class I molar and canine relationship on both sides. The overjet was 3.1 mm, and the overbite was 2.7 mm. The patient displayed moderate crowding of both the upper and lower arches (3 mm, and 4 mm, respectively) that could be treated on a non-extraction basis. A cone-beam computed tomography (CBCT) apparatus was used (Pax-i3D; Vatech, Yongin, Korea) to acquire an image before treatment. This revealed no root resorption, dental abnormalities, or traumatic or pathologic lesions in the alveolar crests.

Treatment objectives and treatment alternatives

The objective of the treatment was the resolution of crowding of both arches without extraction using enamel reduction through interproximal stripping. Four treatment options were considered for this patient: (1) labial orthodontic appliance with metallic brackets; (2) labial orthodontic appliance with ceramic brackets; (3) clear aligners; or (4) lingual orthodontic appliance. The patient was not happy with the idea of using metallic and ceramic brackets because she desired totally invisible orthodontics. She also refused the use of clear aligners because she was hesitant to adhere to the long periods of waring hours of the removable appliances. Therefore it was decided to choose the lingual orthodontic appliance as the treatment modality.

Treatment sequence and 3D imaging acquisition of the teeth

Impressions of the upper and lower arches were taken using alginate (Cavex Impressional; Cavex Holland BV, Haarlem, Netherlands). The impressions were disinfected and poured with elite dental stones. Accurate scanning of the plaster castes is performed with a high-resolution optical 3D scanner (Identica Hybrid; MEDIT, Seoul, Korea) with a precision of ±7 µm.

After the scanning procedure, the stereolithographic files were stored as a standard tessellation language (.stl) file format. Then the files were transformed into digital models using 3Shape Ortho Analyzer software, the upper and lower arches were aligned, and a 3D prediction was made. After the completion of the alignment, the setup was exported to a 3D printer.

The HIRO system for the indirect bonding technique was chosen since it is one of the most accurate manual methods in the positioning of the lingual brackets and significantly reduces transfer errors that occur with other laboratory techniques such as TARG and CLASS [2]. However, a significant modification to this system was introduced by our team, as described below.

Laboratory procedures and the fabrication of the transfer trays (caps)

**Figure 2:**
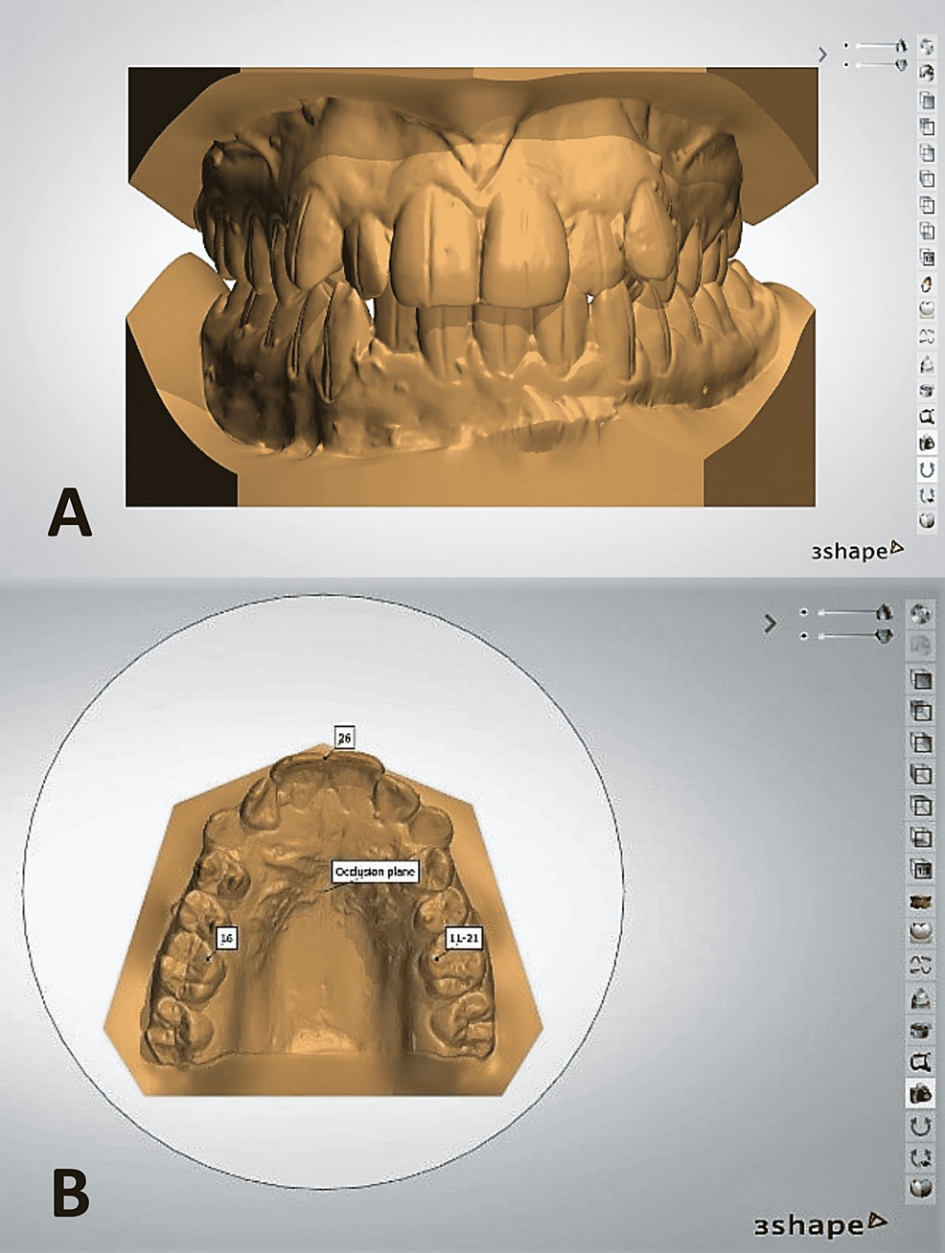
Creation of the bases of the dental models (A), and betting up the occlusal plane on the maxillary virtual model (B)

**Figure 3:**
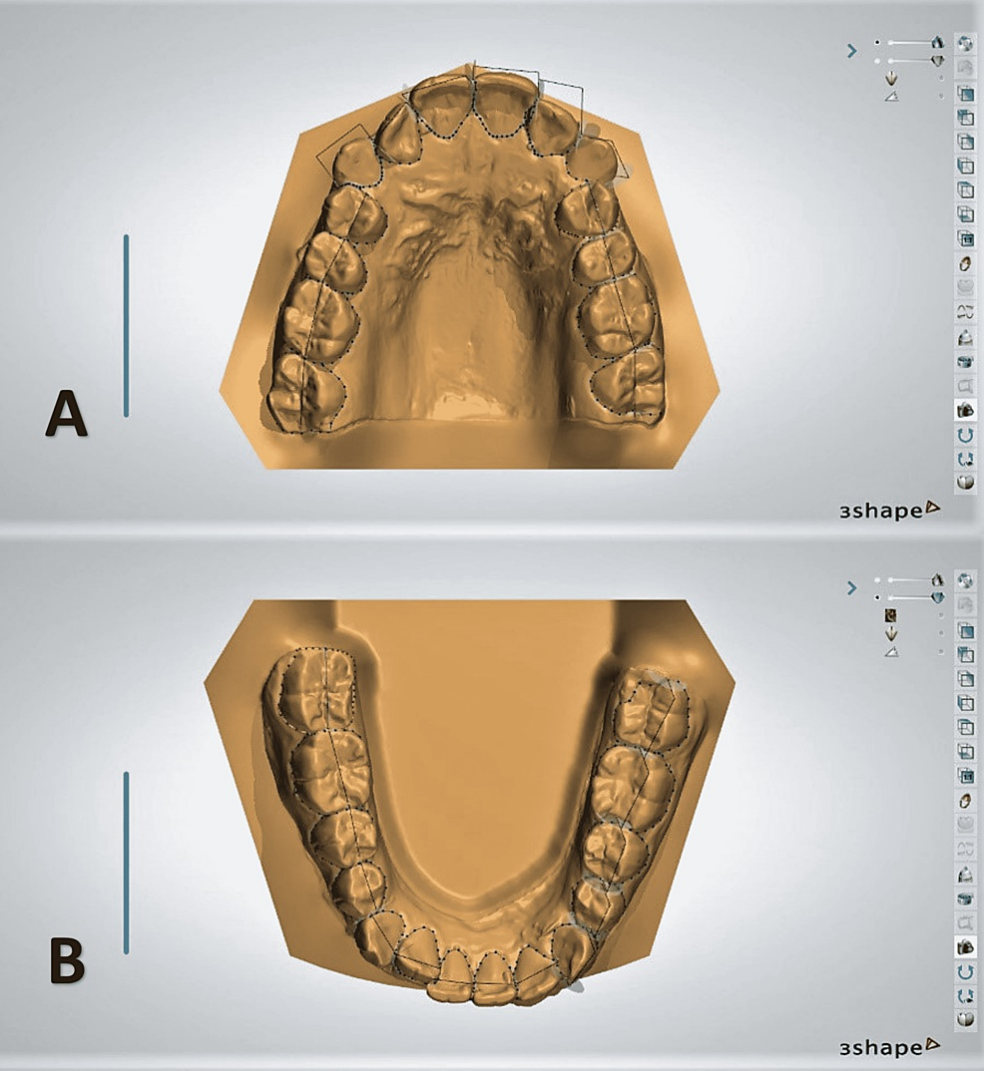
Segmentation in the upper (A) and lower (B) models

**Figure 4:**
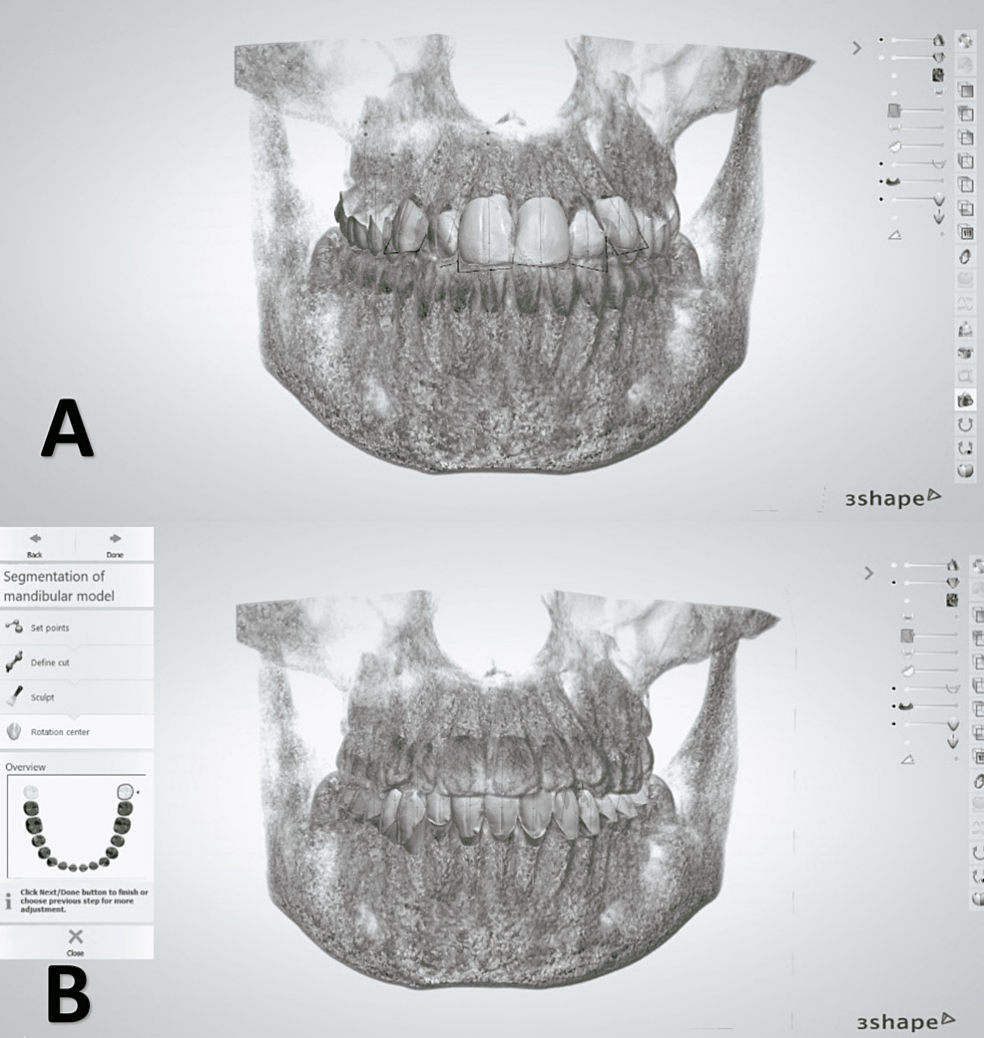
With the help of the anatomical information derived from the CBCT data, the long axis for each tooth is determined (A), and the center of rotation is located (B)

Firstly, the occlusion plan is set on the scanned setup, and the bases of the upper and lower casts were trimmed parallel to this plane (Figure 2). The upper and lower arch forms were obtained, and each of the individual teeth was semi-automatically segmented, keeping the crown anatomy in the medium and the middle-edge directions, making sure that each section extends directly through the contact point (Figure 3). The axis of each tooth and the center of rotation were determined depending on the CBCT image (Figure 4).

**Figure 5:**
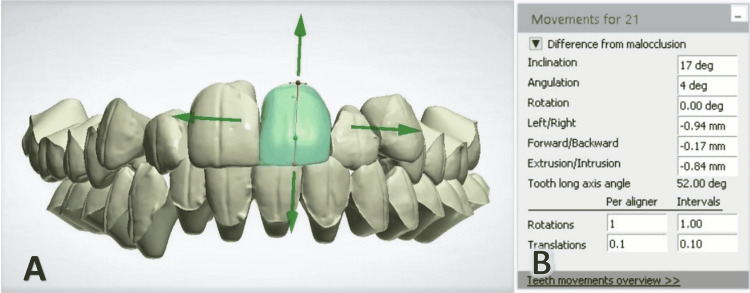
Any tooth can be selected and positioned in 3D. Here, the upper left central incisor was selected (A). Movements can be done in three directions, and rotation can be accomplished around any spatial axis. All the movements and rotations are recorded and displaced (B)

In the original method described by HIRO, the teeth are aligned, and the information added to the brackets is given only by adjusting the axes of the teeth on the setup model without a reference method for identifying torque and tip values. Therefore, an amendment was made by two of the co-authors (JMKB and TZK) to this technique where the McLaughlin-Bennett-Trevisi (MBT) prescription was used to give the torque and tip values. The teeth can be individually chosen and manipulated in the three dimensions of space by clicking on the specified tooth and rotating it in any direction to achieve the desired tip and torque. The procedure can be repeated for the rest of the teeth until the final shape of the arch and the occlusion is achieved (Figure 5).

**Figure 6:**
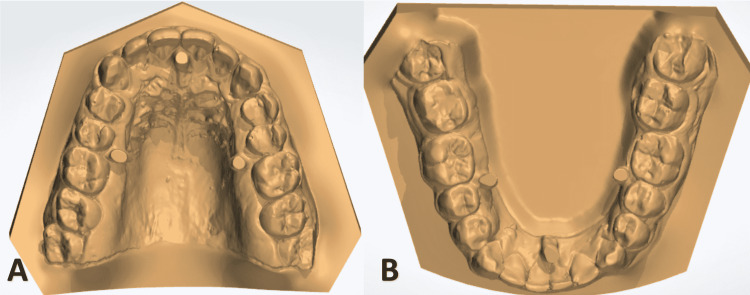
Upper (A) and lower (B) supporting posts at three points

In the original HIRO method, three posts are installed within the wax after completing the fabrication of transfer trays; two posts in the posterior region and one in the anterior region. These posts aim to maintain the vertical height of the basic archwire that may be used later when a repositioning of a bracket is required. Therefore, a modification was also added by two of the co-authors (JMKB and TZK) to standardize the position of the wire height if there was a need to reposition a bracket again. In the proposed modification, the three posts were replaced by determinants that were digitally designed and printed with the final model (Figure 6).

Finally, the teeth were moved to the final positions; then, the models were evaluated on a digital grid which allows for checking the bilateral symmetry of the arches and occlusal interferences for any minor adjustments required. After that, the final digital setup 3D images are saved as a new setup (.stl) file, and the 3D printing of the virtual setup is commanded to begin the placement of the lingual brackets. This is done by the Fused Deposition Modeling (FDM) technique that consists of a movable head, which deposits a thread of molten medical-grade acrylonitrile butadiene styrene (ABS) material on the substrate.

**Figure 7:**
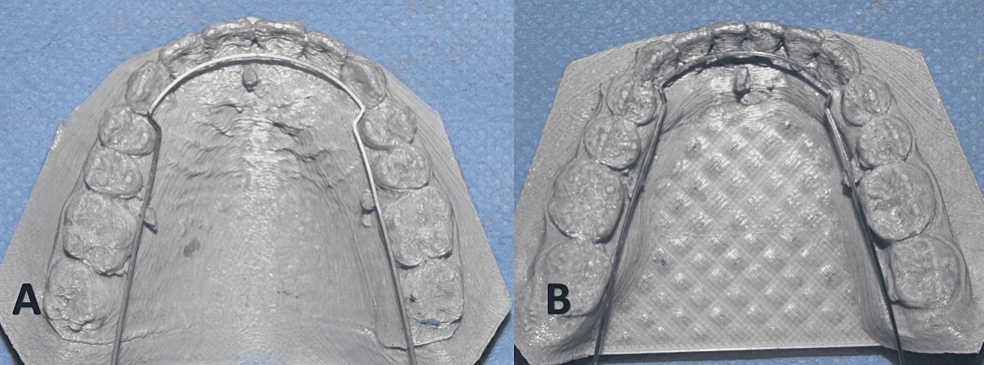
The basic archwire used to hold the brackets into their places A: The upper jaw, B: The lower jaw

Lines were drawn in the gingival third of the palatal surfaces of the upper teeth and in the middle third of the lingual surfaces of the lower teeth to locate the positions of the lingual brackets. These lines were drawn to help in placing the lingual brackets properly, keeping a distance of 1.5 mm between the gingival edges of the brackets and the gingival margins. As in the HIRO® system, the basic archwire used to determine the vertical height of the lingual brackets was made up of stainless steel and had a diameter of 0.017" × 0.025". Care was given to be placed as close as possible to the lingual surfaces of the teeth (Figure 7). 

**Figure 8:**
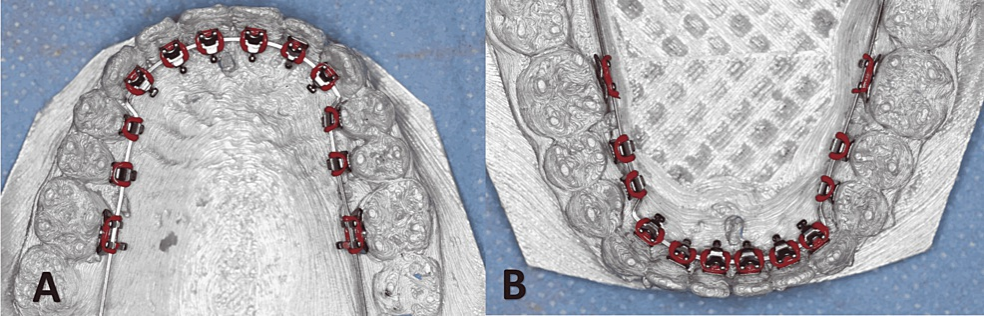
The lingual brackets are placed at their ideal positions and ligated to the basic stainless steel archwire in the upper (A) and lower arch (B)

The six anterior brackets were placed first by attaching them to the basic wire so that the distance between the bracket base and the lingual surface was minimal to reduce the thickness of the composite pad as much as possible; then, the premolars brackets were positioned after adapting the basic wire making sure that each bracket was placed in the center of the lingual surface of the tooth (Figure 8).

**Figure 9:**
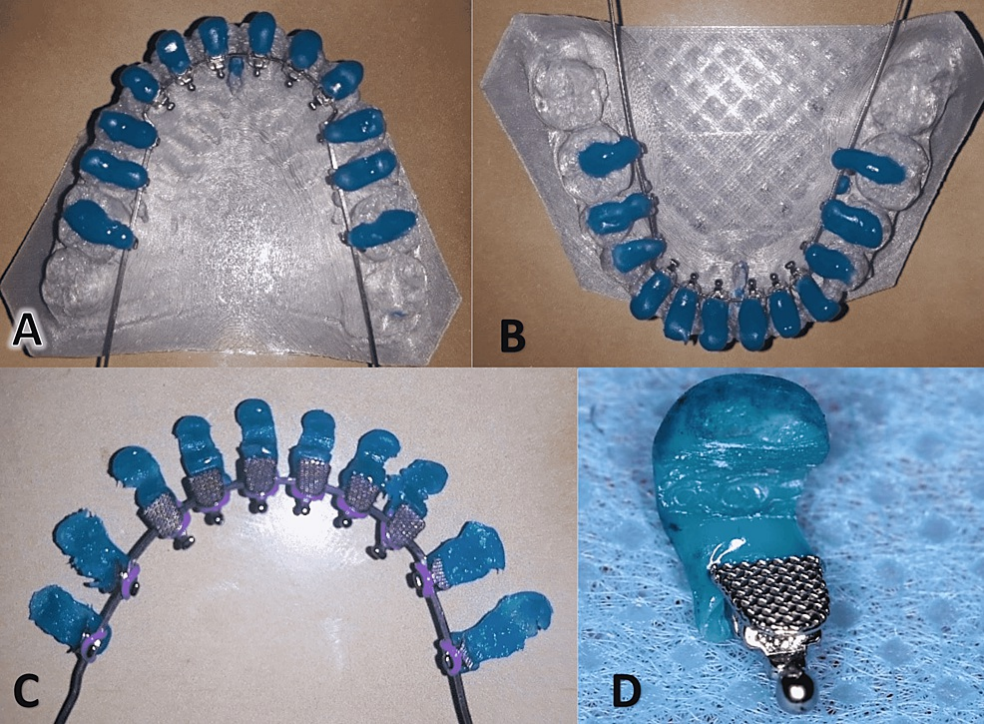
Individual transfer caps are fabricated for each tooth A: Upper dental arch. B: Lower dental arch. C: The lingual brackets being engaged to the basic archwire and the transfer caps are already bonded to the brackets. D: An enlarged view of one bracket transfer cap.

After isolating the setup cast in silicate, the individual transfer cap for each bracket was manufactured using a light-cured blue composite (Resilience®; Ortho Technology, Lutz, Florida,). During transfer cap building-up, care was taken to extend the caps to the incisal third of the bracket and cover at least 2 mm of the buccal surface (Figure 9).

**Figure 10:**
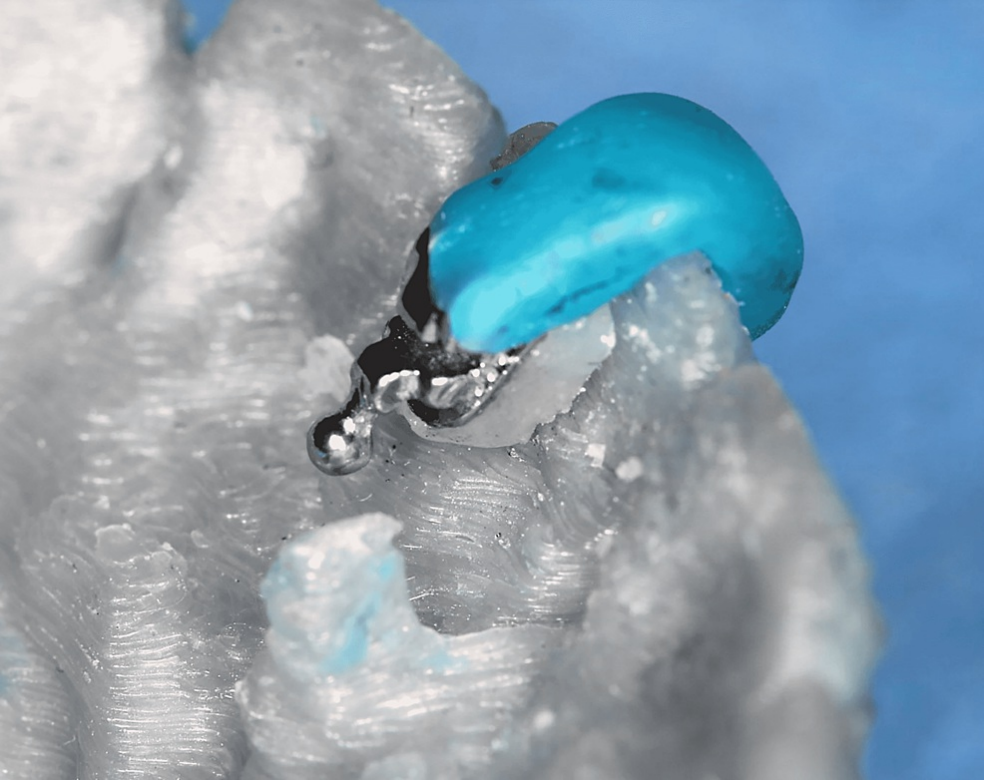
The composite pad is fabricated to lay between the mesh of the lingual bracket and the lingual surface of the tooth. The accuracy of this composite pad is important to ensure proper positioning of the bracket intra-orally

**Figure 11:**
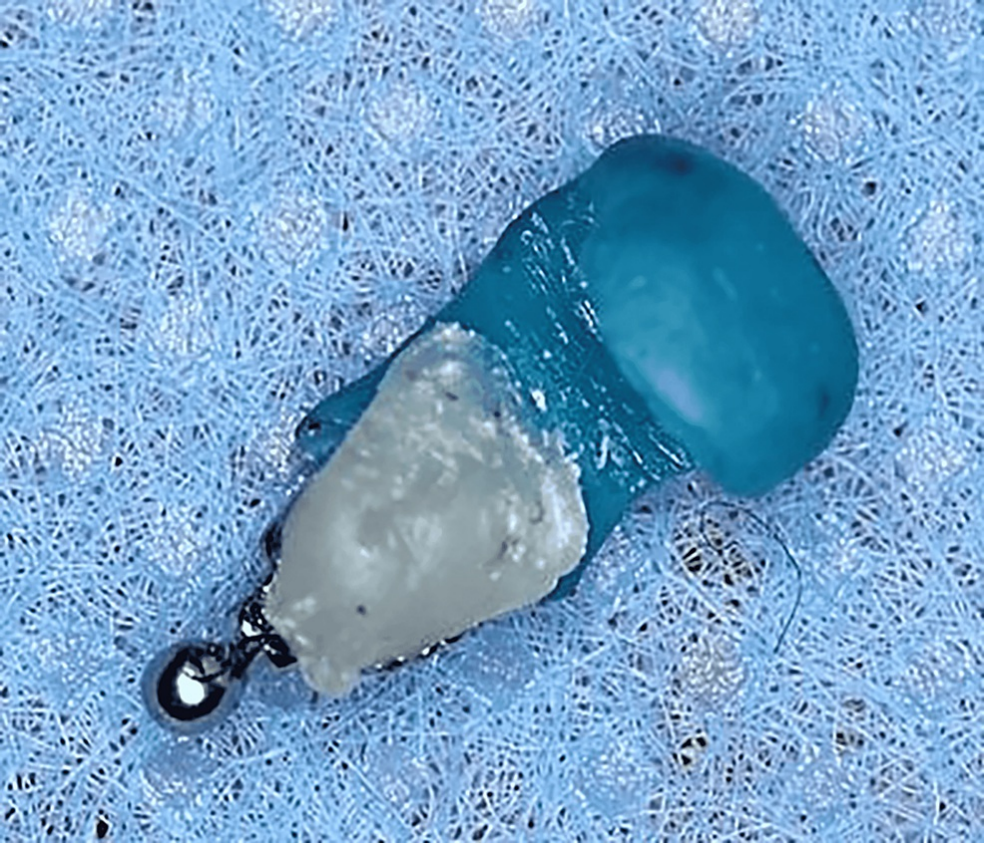
The lingual bracket with the transfer cap and the attached composite pad ready to be transferred to the specified tooth inside the mouth

Each bracket's base was cleaned with acetone, then a light-cured composite (Light Bond™; Reliance Orthodontic Products, Inc., Itasca, Illinois) was added to the base of each bracket after being returned to the corresponding tooth on the printed model and was light-cured. At this step, each bracket had its own pad to fill in the space between the bracket and the lingual surface of the tooth (Figure 10). Finally, the bracket became ready to be transferred to its position in the patient's mouth using the manufactured transfer caps (Figure 11).

Intraoral bracket bonding

**Figure 12:**
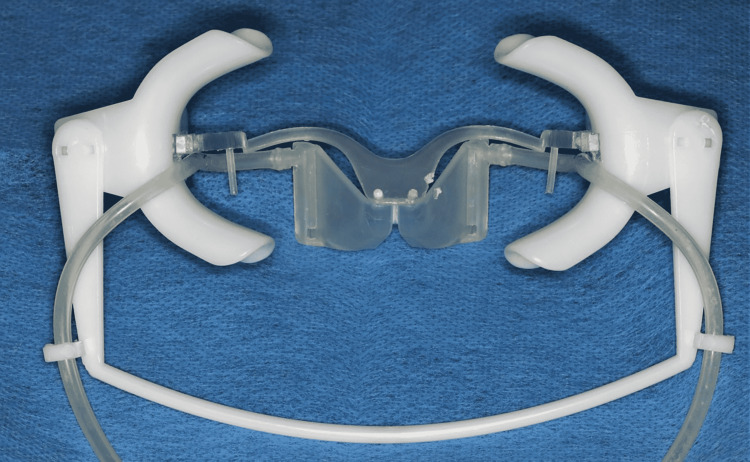
The Nola Dry Field System used in the current case

The bonding surface of the bracket pads was cleaned with acetone to remove all contaminants and leave the composite bonding surface clean. The lingual tooth surfaces were cleaned using a polishing paste (Super Polish®; KerrHawe SA, Bioggio, Switzerland) and a rubber cup mounted on a low-speed rotary instrument. The tooth was washed with water and dried with a stream of oil-free air. Isolation was controlled using Nola Dry Field System (Great Lakes Orthodontics, Tonawanda, New York) and cotton rolls (Figure 12). The lingual surface of the tooth was etched with 37% orthophosphoric acid gel (3M Scotchbond etchant; 3M Dental Products, St. Paul, Minnesota) for 30 seconds and dried with a moisture-and-oil-free source to obtain a uniform and frosty white appearance for another 20 seconds [22]. A layer of bond (Excite®; Ivoclar Vivadent, Liechtenstein, Switzerland) was applied on both the bracket composite pads and etched lingual surfaces, then a small quantity of adhesive resin (Light Bond™; Reliance Orthodontic Products, Inc., Itasca, Illinois) was applied over the bracket pads. The transfer tray is immediately transferred to the mouth and pressed against the enamel in its correct position and removed of excess adhesive, and light-cured for 30 seconds. The transfer trays were removed by diamond burs.

Treatment stages for this patient

**Figure 13:**
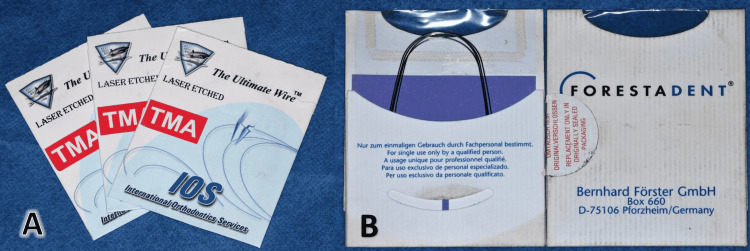
Examples of the archwires used in the current work A: Titanium molybdenum alloy (TMA) archwires, B: nickel-titanium archwires

**Figure 14:**
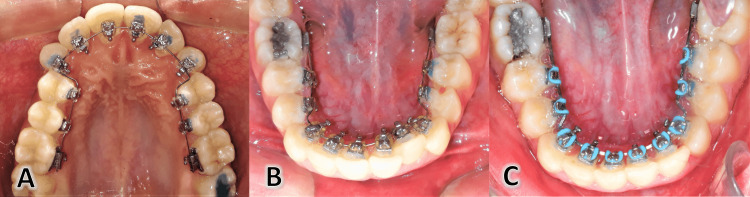
Compensating for the offset between the second premolar and the first molar by changing the thickness of the second premolar composite pad to avoid the need for wire bending in this region A: A soft archwire was used at the beginning of treatment, B: in the intermediate stages of treatment, C: at the end of treatment.

Orthodontic treatment progress included 0.012-inch, 0.014-inch, and 0.016-inch nickel-titanium archwires (Titanol™, Forestadent® Co, Pforzheim, Germany), followed by 0.016×0.022-inch titanium molybdenum alloy (TMA) archwires (The Ultimate Wire™, International Orthodontics Services, Inc., Stafford, Texas), then 0.016×0.022-inch stainless steel (SS) archwires, and 0.017×0.025-inch SS archwires (Figure 13). The archwires were mushroom-shaped to compensate for the offset between the canine and the first premolar on the lingual side; however, the offset between the second premolar and the first molar was compensated during composite pad fabrication instead of using additional wire bending at this region (Figure 14).

**Figure 15:**
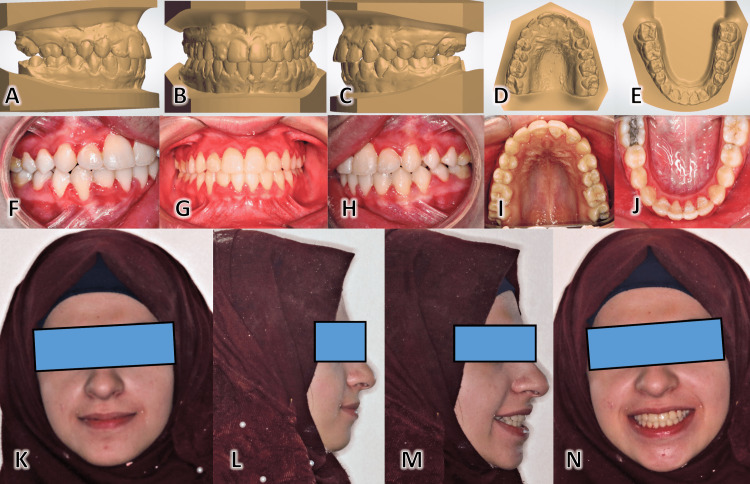
Post-treatment patient records The plaster study models are shown from the right-hand side (A), the frontal view (B), the left-hand side (C), the occlusal view for the upper dental arch (D), and the occlusal view of the lower dental arch (E). Intra-oral photographic images of this case show the dentition from the right-hand side (F), the frontal view (G), the left-hand side (H), the occlusal view for the upper dental arch (I), and the occlusal view for the lower dental arch (J). Extra-oral photographic images included the following poses: the frontal face image at rest (K), the lateral image at rest (L), the 45-degree image with a smile (M), and the frontal face image at smile (N).

The patient was followed up every month. Archwires were only replaced when there was an improvement in teeth alignment, and the following archwire could be inserted with a minimal amount of bending and without exerting excessive force on teeth. A gentle interproximal reduction was carried out from canine to canine to create the required space for aligning the crowded teeth using single-sided hand-held metal abrasion strips (Steelcarbo® strips; Horico, Berlin, Germany). The brackets were removed, and the teeth were polished. Upper and lower removable splints were placed to guarantee post-treatment stabilization. The case at the end of treatment can be seen in Figure 15.

## Discussion

Lingual appliances have become a viable alternative to the successful treatment of most adult and adolescent patients who seek completely invisible appliances [2]. These appliances are of special importance for patients' physical appearances as well as they have a better effect on the patients' oral-health-related quality of life in comparison with other fixed appliances [23].

Although the HIRO system is considered one of the most widely used techniques because of its ease and accuracy, however, this system is technically susceptible to errors as it is completely manual and is affected by the manual dexterity and expertise of the operator [10]. Computer technology has progressed over the years to allow for accurate 3D scanning, printing, and simulation to improve the effectiveness of orthodontic treatment [24]. Also, CBCT has become an important imaging modality in orthodontic diagnosis, treatment planning, and evaluation of clinical outcomes [25]. Therefore, to enhance the reliability, precision, and feasibility of lingual orthodontics, there is a great demand for employing 3D digital technology in the process of fabrication of these appliances [26].

The current case report shows that the proposed method has several advantages. First, accuracy of the setup in achieving the desired tip and torque for each bracket in the whole dental arch without being affected by any mistakes that could be overlooked by the eyes of the operator. In addition to the accurate placement of the brackets, there is also good control over the length and width of the dental arch at the end of the treatment [27]. Second, the planning procedure is easy to accomplish on screen and does not require any specialized laboratory equipment. A huge save in time can be achieved in comparison with the manual positioning of teeth in the ordinary laboratory steps with the embedded back and forth movements and trials. Besides, the practitioner has the freedom to choose any type of lingual brackets, especially those with a low profile that has been shown to be more comfortable for patients [28]. Third, the patient can see the final result onscreen and share in the decision on the final treatment plan. Finally, it is also a cost-effective method for patients who cannot afford to pay for fully customized lingual appliances that are usually accompanied by high costs.

## Conclusions

In this case report, the modified HIRO system of indirect bonding of lingual orthodontics through merging digital technology in model setup and 3D tooth positioning with the manual steps of bracket placement and transfer is an accurate method of moving the teeth into their desired positions, achieving the goals of orthodontic treatment. The proposed technique is simple, does not require expensive devices and materials, is cost-effective, and is suitable for routine use in orthodontic clinics.
